# Examining uptake of online education on obstructive sleep apnoea in general practitioners: a randomised trial

**DOI:** 10.1186/s13104-016-2157-5

**Published:** 2016-07-19

**Authors:** Christine Paul, Shiho Rose, Michael Hensley, Jeffrey Pretto, Margaret Hardy, Frans Henskens, Tara Clinton-McHarg, Mariko Carey

**Affiliations:** School of Medicine and Public Health, The University of Newcastle, University Drive, Callaghan, NSW 2308 Australia; Department of Respiratory and Sleep Medicine, John Hunter Hospital, Lookout Road, New Lambton Heights, NSW 2305 Australia; School of Electrical Engineering and Computer Science, The University of Newcastle, University Drive, Callaghan, NSW Australia; Hunter Medical Research Institute, New Lambton Heights, NSW 2308 Australia; Woolcock Institute of Medical Research, 431 Glebe Point Rd, Glebe, NSW 2307 Australia; GP Education Committee, Australasian Sleep Association, Blacktown, Australia

**Keywords:** Australia, General practice, Education, Professional, Sleep apnea, Obstructive, Sleep disorders

## Abstract

**Background:**

Obstructive sleep apnoea (OSA) affects up to 28 % of the adult population in Western countries. The detection and management of OSA by general practitioners (GPs) can be poor. The study aimed to examine what influence enhanced invitations had on uptake of on-line learning modules for OSA by GPs, and whether recent referrals of patients to sleep specialists influenced uptake.

**Methods:**

Practicing GPs in regional Australia were identified and randomised to receive either an enhanced or standard invitation letter to a new on-line education module for OSA. The enhanced letter included indication that the module was eligible for professional accreditation and described the prevalence and burden of sleep disorders. Some included extra emphasis if the GP had recently referred a patient for diagnostic investigation of OSA. Two reminder letters were sent.

**Results:**

Of 796 eligible GPs who received the letters, sixteen (2 %) accessed the website and four completed the modules over the four-month study period. GPs who received an enhanced invitation letter were not significantly more likely to access the website compared to GPs who received the standard invitation letter. Recent referral of a patient for diagnostic investigation was also not a significant factor in influencing use of the module.

**Conclusion:**

GP interest in on-line education about OSA appears low, and emphasis of relevant recent past patient(s) and the opportunity for professional education points was not successful in increasing engagement. There is a need to identify effective approaches to improving the detection and management of OSA in general practice.

## Background

Obstructive sleep apnoea (OSA) is a common sleep disorder. It is estimated to affect up to 26 % of the adult population in the United States, with 10 % of overall cases considered moderate or worse [1]. OSA is more prevalent in men, older adults and those with a higher body mass index [1]. The condition is characterised by the repetitive collapse of the upper airway during sleep, resulting in frequent arousals and poor sleep quality. OSA can elevate the risk of mortality and morbidity, with reduced daytime vigilance impacting on motor vehicle and occupational accidents [2, 3]. Studies have also shown that OSA leads to hypertension [4, 5], cardiovascular disease, stroke and heart failure [4, 6–8]. The economic burden of OSA is significant in terms of health-related care; other medical conditions that occur as a result of OSA; and indirect costs such as loss of productivity [9].

The general practice setting (i.e. family care or primary care) is central to the detection of sleep disorders, given that 30 % of general practice patients report symptoms of sleep difficulty [10] and a high proportion of the population attend general practice in a 12 month period [11, 12]. However, the limited available data suggests OSA detection rates are low in general practice [13]. In addition to detection, the general practice setting is integral to ongoing care and management of OSA. While specialist advice may be required for formal diagnosis and for initiation of some therapies, the monitoring of outcomes such as the use of continuous positive airways pressure treatment [14, 15] or maintained weight loss [5, 16] is the role of general practice in some countries.

As the accurate detection and management of sleep disorders is a complex task, it is likely that general practitioners (GPs) are not as well skilled in this field as they may like to be [17–19]. GPs may see only a small number of new sleep disorder cases in any given year, and may not be up-to-date with recent evidence. On-line education can provide timely access to current information, links to relevant resources, enhance knowledge and improve performance of the user [20]. On-line approaches have been found to be just as effective as other approaches for providing continuing medical education [21]. However, an issue of crucial importance to the effectiveness of such approaches is that of acceptability, which is reflected in uptake, or the degree to which a tool or resource is accessed by the target group. The limited research regarding the uptake of on-line learning opportunities by GPs suggests uptake rates are generally low across a range of disease states [22–24] and indicates a need to explore how low uptake rates might be addressed.

The purpose of this study was to examine whether an enhanced invitation letter which emphasised recent past patient diagnoses (compared to a basic invitation letter) would improve GP engagement with an on-line education module on OSA in a sample of Australian GPs. The study also explored whether recent patient referrals to sleep specialists was related to uptake of the on-line learning module regardless of which invitation letter was received.

## Methods

### Sample and procedure

GPs practicing in the Hunter and New England area of New South Wales (NSW), Australia were eligible to participate in the study. Practicing GPs in the Hunter and New England regions were identified via an online medical database (Medical Directory of Australia) and randomised to receive either an enhanced (intervention) or standard (control) invitation letter. All currently-practising GPs were eligible for the study. Randomisation was stratified based on whether or not each GP had a patient referred to the Newcastle Sleep Disorders Services (who provide all public sleep-related clinical services in the Hunter and New England regions). An optimal allocation ratio of 1:1 was used. Standard web monitoring tools embedded within the site stored data regarding participant’s website use, in de-identified form. Invitation letters were mailed from November 2013 to January 2014. Up to two reminder letters were sent at fortnightly intervals to GPs who had not accessed the on-line module.

### On-line learning module

The on-line module was developed as a learning tool for the identification and management of OSA in the general practice setting. Evidence-based content was drawn from the Sleep Health Foundation, the Australasian Sleep Association, relevant government sites, and input from experts in the field of sleep health (MH and JP). A consensus group of sleep physicians, GPs, practice nurses and behavioural scientists agreed on the key content for the learning modules. The modules included current evidence-based guidelines; important clinical issues; and links to useful resources for OSA. The on-line module was presented as an interactive learning tool and provided the opportunity to reflect on learning needs. Throughout the module, users were presented with patient-based scenarios with accompanying questions to assess their understanding. The module was divided into five sections: (1) general background; (2) examination/history taking; (3) diagnosis; (4) treatment; and (5) on-going management in the general practice setting. The module could be completed over several sessions, took approximately 6 h to complete and was hosted by the Hunter Postgraduate Medical Institute which provides a range of accredited educational activities for GPs in the state of NSW. When accessing the site for the first time participants were asked about reasons for participation and previous internet use for educational purposes. On completion of the learning module participants were invited to complete a short evaluation survey.

### The invitation letters

*Intervention* (*enhanced letter*) The enhanced letter provided instructions on how to create log-ins and access the on-line module. If the GP had referred a patient to the Newcastle Sleep Disorders Services for a suspected sleep disorder within the last 2 years, the letter also highlighted this to the recipient to personalise the invitation and emphasise the relevance of the module. The letter also informed of the opportunity to redeem professional development points recognised by the Royal Australian College of General Practice by completing the module. In Australia, acquiring such ‘points’ is necessary for a range of reasons including renewal of medical registration, eligibility for government healthcare rebates and practice accreditation. The prevalence and burden of sleep disorders were also described. Evidence-based strategies for effective communication were adopted, such as simple language and sentence structure, repetition of main points, and letter design to maximise uptake to the website. Participants who did not access the module were sent reminder letters at 2 and 4 weeks later.

*Control* (*standard letter*) The standard letter informed the recipient of the availability of the module and provided instructions on how to create log-ins and access the module. Participants in this arm were also eligible to receive the same professional development points detailed in the enhanced letter, however this letter did not inform the recipient of this opportunity.

### Reasons for participation and prior internet use

All GPs who accessed the module were asked to indicate their reasons for participating from a list of possible options (“The offer of professional development points”; “I have patient(s) who have been diagnosed with OSA”; “I am interested in learning about OSA in the general practice”; “Other”) and whether they had made prior use of on-line resources for professional education.

### Statistical analysis

Descriptive statistics (means and proportions) were used to describe data regarding uptake. The Chi square test of independence and a 5 % significance level were used to make comparisons between the standard versus enhanced letter groups and GP prior referral/non referral to the Newcastle Sleep Disorders Centre. Reasons for participation were reported as frequencies.

## Results

**Fig. 1:**
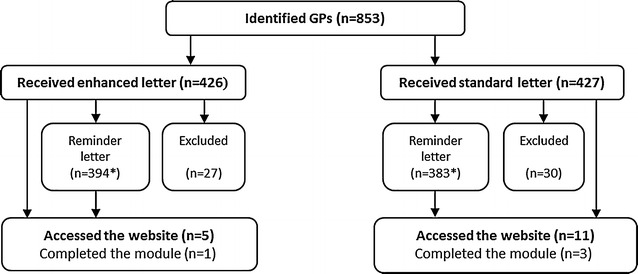
Outline of invitation letter randomisation and subsequent number of GPs who accepted the invitation and/or completed the OSA online learning module. 853 GPs were identified from the Medical Directory of Australia database and randomised to receive either an enhanced letter containing details of the on-line modules eligibility to receive professional accreditation, or a standard letter inviting the GP to access and complete the module. After reminder letters were sent, 5 GPs that received the enhanced letter accessed the website with 1 completing the learning module. 11 GPs that were sent the standard letter accessed the website with 3 completing the learning module. *Asterisk* indicates participants who logged into the module within the first 2 weeks did not receive a reminder

A total of 853 GPs were identified from 295 different practices. Figure 1 illustrates the process of recruitment, randomisation and uptake. Fifty-seven GPs were excluded as they were no longer practicing at their identified site (invitation letters were returned to sender), leaving 796 GPs for analysis. Sixteen (2 %) accessed the module and four completed the module over the four-month study period. Of those who accessed the module and responded to embedded survey items (n = 11), all indicated previous Internet use for education purposes or information seeking. GPs who received an enhanced invitation letter were not significantly more likely to access the module compared to those receiving the standard invitation letter (Table 1). Recent referral of a patient for diagnostic investigation of OSA (404/796—50.8 % of GPs) was also not a significant factor in influencing uptake of the module (Table 2).

**Table 1:** Uptake of module according to invitation letter allocation

	Intervention	Control	P value
Accessed module (log-in), *n* (%)	5/399 (1.3 %)	11/397 (2.8 %)	>0.05
Time taken to log-in (days), mean ± SD	41.0 ± 21.9 (range 25–78)	30.9 ± 15.7 (range 7–71)	>0.05
Sections accessed, mean ± SD	1.6 ± 1.9 (range 0–5)	1.7 ± 2.1 (range 0–5)	>0.05
Time spent on module (minutes), mean ± SD	103.2 ± 151.6 (range 22–372)	85.8 ± 93.3 (range 17–277)	>0.05

**Table 2:** Uptake of module according to previous patient referral to sleep clinic

	Previous referral	No referral	P value
Module log-in, *n* (%)	7/404 (1.7 %)	9/392 (2.3 %)	>0.05
Time taken to log-in (days), mean ± SD	37.4 ± 16.0 (range 25–71)	31.4 ± 19.5 (range 7–78)	>0.05
No. sections accessed (out of 5), mean ± SD	1.7 ± 2.3 (range 0–5)	1.7 ± 1.9 (range 0–5)	>0.05
Time spent on module (minutes), mean ± SD	96.3 ± 124.6 (range 22–372)	87.3 ± 104.0 (range 17–277)	>0.05

### Reasons for participation

Of the four GPs that completed the online learning module, three indicated that they were motivated to participate by interest in OSA in the general practice setting. The remaining GP cited the offer of professional development points.

## Discussion

The study findings indicated a low level of interest in using web-based learning in relation to OSA, given only 2 % of the sample logged into the study module. An enhanced invitation letter was not effective in promoting uptake for on-line learning in the sample of GPs. The proportion of GPs who accessed the module was similar to some previous studies examining GP participation in online education [25, 26], however other studies have reported substantially greater uptake levels [27, 28]. Studies with higher participation rates appeared to incorporate intensive multi-modal recruitment strategies. For example, one study sent up to 33 email contacts over a 45-week period in order to achieve a 45 % rate of engagement (measured by log on and completion of one module) for a GP sample in the United States of America [27]. Such a high level of persistence is not generally permissible by human research ethics committees, so was not able to be included as part of this study. A systematic review of strategies for improving GPs’ survey response rates reported that sequential mixed mode approaches (such as an online survey followed by a paper survey with the reminder) was more effective than single strategies or combination of strategies sent simultaneously [29].

The study results regarding the lack of effect of the enhanced invitation letter compared to a standard letter should be interpreted with caution—in that only a small proportion of the sample accessed the module, limiting the power of the study to find a significant difference between the two experimental groups. It seems likely that factors other than the content of the letter (e.g. GP time constraints, mode or frequency of contact, lack of salience of OSA) may be more likely to have an effect on GPs’ engagement with learning resources for OSA. While the appearance of the recruitment letters may have played a role in the low participant uptake, a recent study [30] found a conventionally worded invitation letter was more successful in recruiting volunteers than a letter that used motivational language or was enhanced with imagery and graphic design due to the plain letter being perceived as more professional and ‘credible’.

If indeed the first of the two main components of the enhancement—reference to previous patients suspected of OSA—did not influence module uptake, the data suggest that the idea of a ‘teachable moment’ is not applicable in this instance. This may be due to the time lag between the referral of the patient and receipt of the letter. There is also a possibility of dilution of effect in that only half of the GPs in the study had made a referral to the sleep clinic in the prior 2 years. Perhaps an immediate invitation very closely timed to patient referral may act as a more effective impetus for further learning about OSA. The second component- the availability of professional education points- did not appear to encourage website uptake. This is consistent with other studies on practice recruitment [31].

The topic of OSA may register as a low learning priority for GPs in general, perhaps because it does not have immediate short-term health implications for most patients. There are a wide variety of opportunities open to GPs for gaining professional development points, although few are available in relation to OSA. The high degree of choice reduces the chance that any one opportunity (such as the OSA learning module) will be taken. It may also be that some GPs believe their level of understanding is sufficient. However, a US study of 105 primary physicians found only 10 % considered their sleep knowledge to be good or excellent, while the remainder rated themselves to be fair or poor [18]. This finding was reflected in knowledge assessment scores from this study, where the average knowledge score was 34 % (range 3–94 %). A focus group conducted with a small sample of Australian GPs [17] indicated participants felt their knowledge of sleep disorders and associated management was fair to poor [17]. Therefore, continued medical education in OSA is likely to be of benefit for a very high proportion GPs. Alternative approaches to increasing knowledge and skills about OSA in general practice includes a sleep “certificate” pathway for GPs provide additional incentive to become subspecialized in sleep care.

GPs appear to be satisfied with online learning techniques [15, 32], and our participants reported prior use of on-line learning. However, a recent survey of 2500 Australian GPs reported that most preferred face-to-face lectures rather than online learning (83 vs. 55 %) [33]. A similar result was found in a survey of rural GPs and their educational preferences regarding diabetes care [34]. Being less familiar with computers or low computer literacy can act as barriers to the use of on-line learning opportunities among GPs and may partially explain the preference for face-to-face learning [35, 36]. Younger GPs have also been shown to have higher regard for on-line education approaches than do older GPs [33]. One of the limitations of this study was an inability to collect personal data such as the age of non-participants. The limited available data suggests that learners are more likely to accept on online course if they perceive it offers a perceived advantage over available non-internet alternatives, is easy to use technically, and compatible with their values and norms [37].

## Conclusions

GP interest in online education about OSA appears low, and enhancement of a written invitation highlighting past patient referrals for sleep disorders and the offer of professional development credit points was not successful in increasing engagement. There is a need to identify effective approaches to improving the detection and management of OSA in general practice.

## Abbreviations

OSA: obstructive sleep apnea
GP: general practitioner
NSW: New South Wales
